# Retrospective study of frequency of ABO and Rhesus blood group among population of Safdarabad and Faisalabad cities of Pakistan

**DOI:** 10.1186/s13104-020-05429-z

**Published:** 2021-01-07

**Authors:** Ali Sabir, Arslan Iftikhar, Muhammad Umar Ijaz, Ghulam Hussain, Azhar Rasul, Rana Khalid Iqbal, Faiqa Sajid, Haseeb Anwar

**Affiliations:** 1 Department of Physiology, Faculty of Life Sciences, Government College University, Faisalabad, Pakistan; 2 Department of Zoology, Wildlife and Fisheries, University of Agriculture, Faisalabad, Pakistan; 3 Department of Zoology, Faculty of Life Sciences, Government College University, Faisalabad, Pakistan; 4 Institute of Molecular Biology and Biotechnology, Bahauddin Zakariya University, Multan, 60800 Pakistan

**Keywords:** ABO blood group, Rh (d) phenotypes, Blood group frequency

## Abstract

**Objective:**

The current study aimed to investigate the ABO and rhesus (Rh) blood group frequency in the people of District Faisalabad and Sheikhupura, Punjab Province, Pakistan. The retrospective study was conducted on more than thirty thousand people including both male and female patients admitted to the Tehsil Headquarter Hospital, Safdarabad and The Best Hospital, Faisalabad. Blood samples were taken from each subject and subsequently ABO and Rh blood groups were evaluated separately. The antigen antibody agglutination slide test for blood grouping (ABO) and Rh were used to assess the blood group frequencies.

**Results:**

The frequencies of ABO blood group distribution indicated that blood group B was predominant in the people of Safdarabad followed by O, A and AB respectively. While, among people of Faisalabad, blood group O was predominant followed B, A and AB respectively. Rh negative phenotype was found lesser distributed as compared to the positive Rh phenotype.

## Introduction

Throughout the world, the blood is considered a permanent and established identity of an individual. Though the blood group of an offspring is decided by the genetic material inherited from mother and father, even the siblings may have different blood groups, with exception of identical twins and triplets only indicate that blood groups are predisposed to individual genetic makeup [1]. Around 400 blood group systems have been reported till now and among them, the most important are ABO and Rh. ABO and Rh blood group systems are considered as the most crucial since they are crucial in blood transfusion too and are also of great clinical importance by virtue of their relationship with the various hemolytic diseases of newborn [2, 3]. The chromosome 9 and 1 have the genes for ABO and Rh (D) respectively in the human genome.

The ABO blood types were first discovered by the Austrian Physician Karl Landsteiner in 1901. Different sugars and proteins which constitute the blood group antigens are present on the surface of our red blood cells. There are about thirty different types of antigens present on the surface of these red blood cells. The ABO antigens are synthesized well before the birth and persist throughout the whole life. The fetus acquires ABO antibodies passively from their mother before birth, but by three months of age, infants start preparing their own specific antibodies [4]. On the other hand, the Rh blood group system was discovered by Landsteiner and Weiner in 1940. Immunogenicity of the Rh factor together with A, B antigens made it compulsory for pre-transfusion testing [5, 6]. In the Rh blood group system, unlike ABO blood group, D antigen is formed by a group of conformation-dependent epitopes along with the Rh (D) protein [7].

The variation in ABO and Rh blood group distribution exists from one race to another race; all around the world. People of Pakistan also exhibits this variation [8]. It is believed that blood group prevalence has an important role in evolution, genetics research, organ transplantation, and blood transfusion [9, 10]. The current study was aimed to find out the prevalence percentage of ABO blood group type and Rh (D) frequency in the people of Faisalabad and Safdarabad cities of Punjab Province of Pakistan (Additional file 1: Figure S1).

## Main text

### Materials and methods

#### Subjects

A total of 30,682 subjects, including males and females, were screened at Tehsil Headquarter (THQ) Hospital of Safdarabad and The Best Hospital of Faisalabad, Punjab, Pakistan, for blood grouping. Samples were obtained after official permission of competent authorities of hospitals and subsequent verbal or written consent of participants. The samples were collected from the patients presented to above-mentioned hospitals during the period of November 2018 to October 2019. Among these subjects, 15,703 were men and 14,979 were women. Their age was ranged between 16 and 40 years (mean: 27.4 ± 6.2). The majority of study population were presented to hospital for their health screening and blood grouping, which is mandatory for various purposes, such as army recruitment, pre-employment health screening, and screening for driving license etc. Out of 30,682 subjects, 13,477 were from Tehsil Safdarabad, District Sheikhupura and 17,205 were belong to Ghulam Muhammad Abad, Faisalabad. The data were generated and compiled using National Identity Card number as individual identification mark. All data was compiled and recorded in specified perform. The blood samples of the study population were typed by slide method, using ABO and Rh (D) Typing Antisera, Biotec Laboratories®, United Kingdom. Manufacturer’s procedural instructions were followed while experimentation. Results were compared with similar group prevalence studies from neighboring countries. Data were summarized using frequency and percentages.

#### Interpretation of results

Positive: Agglutination; specifies positive reactions to respective subject.

Negative: No agglutination; specifies negative reactions to respective subject (Additional file 2: Table S1).

### Results

The prevalence of ABO and Rh phenotypes in 30,682 subjects (13,477 were of Tehsil Safdarabad District Sheikhupura and 17,205 were from Ghulam Muhammad Abad, Faisalabad) was tested. Amongst the people of Faisalabad, the most common blood group was O (32.78%), followed by blood group B (29.79%), A (22.58%) and blood group AB found at the lowest prevalence (14.83%) (Table 1). Overall, percentage of positive Rh phenotypes and negative phenotypes was 81.01 and 18.99 respectively (Table 1). Additional details for the prevalence of the Rh (D) phenotypes linked with ABO Blood group is mentioned in Table 2.

**Table 1:** Prevalence of the phenotype of ABO and Rh blood groups in Faisalabad and Safdarabad districts

Phenotype	Faisalabad						Safdarabad					
	Men		Women		Total		Men		Women		Total	
	n	%	n	%	n	%	n	%	n	%	n	%
Distribution of ABO blood groups												
A	1965	22.13	1920	23.07	3885	22.58	1500	21.97	1908	28.68	3408	25.28
B	2634	29.66	2493	29.94	5127	29.79	2325	34.06	2232	33.55	4557	33.81
AB	1632	18.38	921	11.10	2553	14.83	600	8.79	364	5.47	964	7.15
O	2647	29.81	2993	35.94	5640	32.78	2400	35.16	2148	32.29	4548	33.74
Total	8878	100	8327	100	17,205	100	6825	100	6652	100	13,477	100
Distribution of Rh blood groups												
Rh (+ ve)	7185	80.93	6753	81.09	13,938	81.01	6150	90.10	5980	89.90	12,130	90.00
Rh (−ve)	1693	19.07	1574	18.91	3267	18.99	675	9.90	672	10.10	1347	10.00
Total	8878	51.60	8327	48.40	17,205	100	6825	50.60	6652	49.35	13,477	100

**Table 2:** Combined distribution of ABO and Rh blood groups in Faisalabad and Safdarabad districts

Blood Groups	Faisalabad						Safdarabad					
	Total participant (n = 17,205)		Male participant (n = 8878)		Female participant (n = 8327)		Total participant (n = 13,477)		Male participant (n = 6825)		Female participant (n = 6652)	
	n	%	n	%	n	%	n	%	n	%	n	%
A Rh- positive	3450	20.05	1614	18.18	1836	22.04	3237	24.02	1425	21	1812	27
A Rh- negative	435	2.52	351	4.00	84	1.00	171	1.26	75	01	96	1.00
B Rh- positive	4407	25.61	2391	27.00	2016	24.21	4119	30.55	2175	32	1944	29
B Rh- negative	720	4.18	243	2.73	477	5.72	438	3.25	150	02	288	04
AB Rh-positive	2232	13.00	1455	16.38	777	9.33	781	5.79	525	08	256	04
AB Rh- negative	321	1.86	177	2.00	144	1.73	183	1.35	75	01	108	02
O Rh- positive	3849	22.37	1725	19.43	2124	25.50	3993	29.60	2025	30	1968	30
O Rh- negative	1791	10.41	922	10.38	869	10.43	555	4.10	375	05	180	03

However, among the people of Safdarabad, the most common blood group was B with prevalence of 33.81% which was slightly greater than blood group O (33.74%). Blood group A percentage was 25.28% and AB shows the lowest prevalence with 7.15% percentage (Table 1). Prevalence of Rh phenotypes were almost same as of Faisalabad; 90% Rh positive and 10% Rh negative phenotype (Table 1). Supplementary details for the prevalence of the Rh (D) phenotypes linked with ABO Blood group is mentioned in Table 2.

### Discussion

Blood group prevalence has huge importance in medical field since it plays an important role in blood transfusion, organ transplantation [11], evolution, and genetics research. It is also associated with various diseases including cardiovascular diseases [12], erythroblastosis in neonates, duodenal ulcer and diabetes [13–15]. In this study, frequency distribution of ABO and Rh blood group among the people of Safdarabad and Faisalabad cities of Pakistan was estimated. The blood group phenotypes among the people of Safdarabad and Faisalabad appeared to be in the B > O > A > AB and O > B > A > AB orders respectively. Among ABO groups, the AB blood group has been reported the least prevalent group all over the world while O blood group is considered the most common blood group in most of the areas. In the current study, amongst the people of Faisalabad, the blood group O was found the most prevailing blood group while blood group B was found the most frequent in Safdarabad city. The findings of O blood group as the most common in Faisalabad city is in accordance with the reported studies of other countries like Saudi Arabia [16], Bahrain [17], Iran [18], India [19], Nigeria [20] and Bangladesh [21]. The findings of Safdarabad is also in compliance with the study conducted in Punjab province of India [6]. Findings in both Faisalabad and Safdarabad are different from the reported studies of Turkey [22] and Palestine [23], where blood group A appeared to be the most prevalent blood group. These variations in the blood group frequency are mainly attributed to the genetic makeup of a particular population living in a particular area.

Although, these variations are when compared on national level, it exhibits a heterogeneity among different cities of Pakistan. These differences can be due to geographical environment, ethnic groups and more specifically due to different sample size. Study from Dir upper [25], Peshawar [26], Sakardu [8] and Dir lower [27] shows that blood group A was the commonest group in those areas. Other studies from twin cities Islamabad and Rawalpindi [28], Gujranwala [29], Lahore [30], Mirpur [31] and Multan [32] reflects the same result like presented in this study of Safdarabad. The current findings of Faisalabad city where blood group O is found the most common is in agreement with the studies conducted in Multan [32], Karachi [33], and Gujrat [34] cities (Table 3).

**Table 3:** Comparison of percentage distribution of ABO and rhesus (Rh) blood groups of Faisalabad and Safdarabad populations with other countries and other cities of Pakistan

Area	A	B	AB	O	Rh +	Rh-	References
Faisalabad	22.58	29.79	14.83	32.78	81.01	18.99	Current study
Safdarabad	25.28	33.81	33.74	33.74	90	10	Current study
Comparison to other countries of world							
Turkey	43.8	16.2	9.2	30.8	86.0	14	[22]
Saudi Arabia	26	18	4	51	92	8	[16]
Bahrain	21.5	24.4	4.5	49.6	94.5	4.5	[17]
Iran	33.1	23.3	8.9	34.7	88.7	11.3	[18]
Palestine	40	22	6	32	97.3	2.7	[23]
India (Punjab)	21.9	37.6	9.3	31.2	97.3	2.7	[19]
Nigeria	24.4	23.8	2.7	48.9	95.6	4.33	[20]
Bangladesh	26.6	23.2	9.6	40.6	96.8	3.2	[24]
Comparison to other cities of Pakistan							
Sialkot	22.2	36.5	9.7	31.34	91.2	8.7	[35]
Islamabad	25.5	33.3	10.0	31.1	91.6	8.4	[28]
Dir upper	32.1	29.8	12.4	25.7	86.4	13.6	[25]
Dir Lower	33.9	27.9	11.3	28.6	92.4	7.5	[27]
Gujranwala	22.9	35.3	9.32	32.4	92.03	7.97	[29]
Peshawar	31.2	31	10.1	27	92.5	7.5	[26]
Lahore	24.2	37.8	9.1	28.8	93	7	[30]
Mirpur	26.3	32.5	31.6	9.4	91.0	9	[31]
Sakardu	30.6	26.8	15.9	26.6	94.8	5.17	[8]
Multan	21.9	36.9	7.33	33.8	92.1	7.83	[32]
Gujrat	18	22	4	56	79.5	20.5	[34]
Karachi	14.4	38.1	8.3	39.2	94.5	5.5	[33]

The frequency of Rh (D) positive and negative phenotypes was 90% and 10% respectively for Safdarabad and 81.01% and 18.99% for Faisalabad. Other studies available in literature archive also indicated somewhat similar pattern in various cities of Pakistan (Table 3) and in different other countries (Table 3).

## Limitations

Our study had few limitations that must be acknowledged. Our results are based on a relatively small sample size that could be improved for developing a more realistic opinion about blood group frequency distribution in the study area.

## Supplementary Information

**Additional file 1: Figure S1.** Map showing study area of Punjab province of Pakistan (Prepared by Authors themselves).

**Additional file 2: Table S1.** Interpretation of results obtained from agglutination test for ABO blood group type.

## Data Availability

All raw data are available from the corresponding author upon request at drhaseebanwar@gcuf.edu.pk.
