# Supplementary figures and images for: Retrospective study of frequency of ABO and Rhesus blood group among population of Safdarabad and Faisalabad cities of Pakistan
Source: BMC Res Notes. 2021 Jan 7;14:12. doi: 10.1186/s13104-020-05429-z (PMC7792172; doi:10.1186/s13104-020-05429-z)

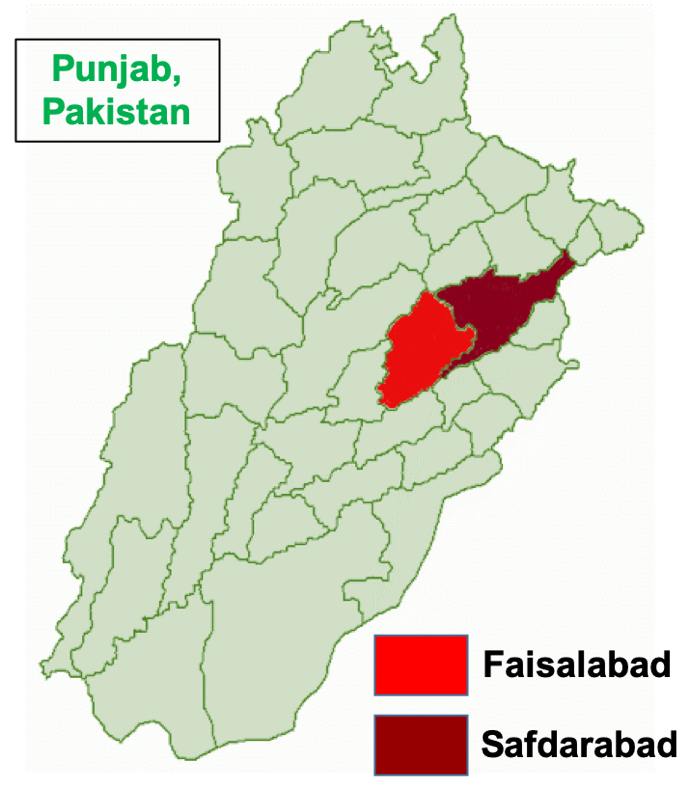


**Figure S1: Map showing study area of Punjab province of Pakistan (Prepared by Authors themselves).**

Supplement: Supplementary file 1 — Additional file 1: Figure S1. Map showing study area of Punjab province of Pakistan (Prepared by Authors themselves). [file 13104_2020_5429_MOESM1_ESM.docx]
