# Supplementary material for: Retrospective study of frequency of ABO and Rhesus blood group among population of Safdarabad and Faisalabad cities of Pakistan
Source: BMC Res Notes. 2021 Jan 7;14:12. doi: 10.1186/s13104-020-05429-z (PMC7792172; doi:10.1186/s13104-020-05429-z)
Supplement: Supplementary file 2 — Additional file 2: Table S1. Interpretation of results obtained from agglutination test for ABO blood group type. [file 13104_2020_5429_MOESM2_ESM.docx]

**Table S1.** Interpretation of results obtained from agglutination test for ABO blood group type.

| **Reaction with anti A** | **Reaction with anti B** | **Blood Group** |
| --- | --- | --- |
| + | + | AB |
| + | - | A |
| - | + | B |
| - | - | O |

**(+) = Agglutination (-) = No Agglutination**
